# Supplementary material for: Modeling glioblastoma heterogeneity as a dynamic network of cell states
Source: Mol Syst Biol. 2021 Sep 16;17(9):e10105. doi: 10.15252/msb.202010105 (PMC8444284; doi:10.15252/msb.202010105)
Supplement: Supplementary file 6 — Source Data for Figure 5 [file MSB-17-e10105-s004.zip › Figure5A_sourcedata/GSEA_3071/nosigdb_stateII.GseaPreranked.1622795151171/RICHARDS_NOWAKOWSKI_SCIENCE_2017_IPC_NEN1_UPREG.html]

Details for gene set RICHARDS\_NOWAKOWSKI\_SCIENCE\_2017\_IPC\_NEN1\_UPREG[GSEA]

|  || Dataset | state23071 |
| Phenotype | NoPhenotypeAvailable |
| Upregulated in class | na\_neg |
| GeneSet | RICHARDS\_NOWAKOWSKI\_SCIENCE\_2017\_IPC\_NEN1\_UPREG |
| Enrichment Score (ES) | -0.5587652 |
| Normalized Enrichment Score (NES) | -3.2967122 |
| Nominal p-value | 0.0 |
| FDR q-value | 0.0 |
| FWER p-Value | 0.0 |
Table: GSEA Results Summary

  

Fig 1: Enrichment plot: RICHARDS\_NOWAKOWSKI\_SCIENCE\_2017\_IPC\_NEN1\_UPREG      
 Profile of the Running ES Score & Positions of GeneSet Members on the Rank Ordered List

  

| PROBE | GENE SYMBOL | GENE\_TITLE | RANK IN GENE LIST | RANK METRIC SCORE | RUNNING ES | CORE ENRICHMENT || 1 | RANBP1 |  |  | 149 | 0.958 | -0.0270 | No |
| 2 | NRG1 |  |  | 356 | 0.708 | -0.0712 | No |
| 3 | COTL1 |  |  | 462 | 0.645 | -0.0906 | No |
| 4 | EZR |  |  | 466 | 0.642 | -0.0843 | No |
| 5 | CORO1C |  |  | 718 | 0.526 | -0.1418 | No |
| 6 | CLMP |  |  | 822 | 0.490 | -0.1624 | No |
| 7 | RND3 |  |  | 860 | 0.482 | -0.1664 | No |
| 8 | SPATS2 |  |  | 989 | 0.447 | -0.1938 | No |
| 9 | NRP1 |  |  | 1372 | 0.362 | -0.2863 | No |
| 10 | EPHB2 |  |  | 1420 | 0.353 | -0.2943 | No |
| 11 | GOLGA8A |  |  | 1526 | 0.335 | -0.3171 | No |
| 12 | ACTB |  |  | 1565 | 0.330 | -0.3231 | No |
| 13 | MAP7D1 |  |  | 1611 | 0.321 | -0.3309 | No |
| 14 | IVNS1ABP |  |  | 1628 | 0.320 | -0.3314 | No |
| 15 | TSPAN5 |  |  | 1861 | 0.291 | -0.3868 | No |
| 16 | CACNA2D1 |  |  | 1878 | 0.289 | -0.3876 | No |
| 17 | CRLF3 |  |  | 1962 | 0.277 | -0.4055 | No |
| 18 | NR2F1 |  |  | 2071 | 0.265 | -0.4299 | No |
| 19 | PTPRS |  |  | 2253 | -0.254 | -0.4728 | No |
| 20 | VASH1 |  |  | 2275 | -0.256 | -0.4753 | No |
| 21 | NEUROD1 |  |  | 2312 | -0.261 | -0.4815 | No |
| 22 | KLF13 |  |  | 2322 | -0.261 | -0.4808 | No |
| 23 | TP53INP1 |  |  | 2337 | -0.263 | -0.4815 | No |
| 24 | PPFIA2 |  |  | 2364 | -0.266 | -0.4851 | No |
| 25 | SEMA6D |  |  | 2407 | -0.272 | -0.4927 | No |
| 26 | CDON |  |  | 2454 | -0.278 | -0.5012 | No |
| 27 | MKLN1 |  |  | 2464 | -0.279 | -0.5004 | No |
| 28 | TMEM158 |  |  | 2476 | -0.280 | -0.5001 | No |
| 29 | NCSTN |  |  | 2487 | -0.283 | -0.4995 | No |
| 30 | SMIM8 |  |  | 2488 | -0.283 | -0.4963 | No |
| 31 | SRGAP3 |  |  | 2508 | -0.286 | -0.4980 | No |
| 32 | BRWD1 |  |  | 2515 | -0.287 | -0.4963 | No |
| 33 | ZNF638 |  |  | 2536 | -0.291 | -0.4981 | No |
| 34 | SORBS1 |  |  | 2639 | -0.307 | -0.5205 | No |
| 35 | CBFA2T2 |  |  | 2646 | -0.308 | -0.5186 | No |
| 36 | GDAP1 |  |  | 2677 | -0.313 | -0.5227 | No |
| 37 | SOGA1 |  |  | 2731 | -0.322 | -0.5326 | No |
| 38 | LRCH3 |  |  | 2747 | -0.326 | -0.5327 | No |
| 39 | CPEB2 |  |  | 2787 | -0.333 | -0.5389 | No |
| 40 | BMPR1A |  |  | 2800 | -0.335 | -0.5382 | No |
| 41 | ZNF302 |  |  | 2809 | -0.337 | -0.5365 | No |
| 42 | PAK3 |  |  | 2839 | -0.343 | -0.5400 | No |
| 43 | ZNF292 |  |  | 2882 | -0.352 | -0.5467 | No |
| 44 | EVL |  |  | 2927 | -0.360 | -0.5539 | No |
| 45 | GTF2IRD2 |  |  | 2946 | -0.364 | -0.5544 | Yes |
| 46 | BSDC1 |  |  | 2961 | -0.366 | -0.5539 | Yes |
| 47 | ATP6V1G1 |  |  | 2968 | -0.367 | -0.5513 | Yes |
| 48 | ZNF286A |  |  | 2986 | -0.370 | -0.5515 | Yes |
| 49 | CPE |  |  | 2994 | -0.371 | -0.5492 | Yes |
| 50 | ATP6V0A1 |  |  | 3014 | -0.378 | -0.5498 | Yes |
| 51 | HIPK2 |  |  | 3029 | -0.379 | -0.5491 | Yes |
| 52 | SCRN1 |  |  | 3051 | -0.386 | -0.5502 | Yes |
| 53 | PAX6 |  |  | 3055 | -0.387 | -0.5466 | Yes |
| 54 | PRKX |  |  | 3093 | -0.397 | -0.5516 | Yes |
| 55 | WDR6 |  |  | 3113 | -0.402 | -0.5519 | Yes |
| 56 | TMSB4X |  |  | 3141 | -0.412 | -0.5542 | Yes |
| 57 | PNISR |  |  | 3160 | -0.416 | -0.5541 | Yes |
| 58 | MIDN |  |  | 3175 | -0.421 | -0.5530 | Yes |
| 59 | NFIC |  |  | 3194 | -0.427 | -0.5528 | Yes |
| 60 | ANKRD10 |  |  | 3195 | -0.427 | -0.5481 | Yes |
| 61 | HEG1 |  |  | 3200 | -0.429 | -0.5444 | Yes |
| 62 | CHRDL1 |  |  | 3219 | -0.432 | -0.5441 | Yes |
| 63 | SOX5 |  |  | 3237 | -0.437 | -0.5436 | Yes |
| 64 | TSPAN6 |  |  | 3240 | -0.438 | -0.5392 | Yes |
| 65 | FYN |  |  | 3262 | -0.444 | -0.5396 | Yes |
| 66 | LDLRAD4 |  |  | 3271 | -0.448 | -0.5367 | Yes |
| 67 | HDAC9 |  |  | 3288 | -0.453 | -0.5357 | Yes |
| 68 | CASC15 |  |  | 3308 | -0.460 | -0.5354 | Yes |
| 69 | SHISA5 |  |  | 3325 | -0.465 | -0.5343 | Yes |
| 70 | NRN1 |  |  | 3326 | -0.466 | -0.5291 | Yes |
| 71 | SYNRG |  |  | 3327 | -0.466 | -0.5240 | Yes |
| 72 | CCSAP |  |  | 3343 | -0.473 | -0.5225 | Yes |
| 73 | MARCKS |  |  | 3369 | -0.487 | -0.5235 | Yes |
| 74 | RPAIN |  |  | 3404 | -0.499 | -0.5265 | Yes |
| 75 | NUDT4 |  |  | 3421 | -0.510 | -0.5249 | Yes |
| 76 | DANCR |  |  | 3428 | -0.511 | -0.5208 | Yes |
| 77 | NOL4L |  |  | 3464 | -0.525 | -0.5238 | Yes |
| 78 | SIPA1L2 |  |  | 3482 | -0.533 | -0.5222 | Yes |
| 79 | SVIL |  |  | 3490 | -0.537 | -0.5180 | Yes |
| 80 | CALCOCO1 |  |  | 3512 | -0.548 | -0.5172 | Yes |
| 81 | ARHGAP21 |  |  | 3538 | -0.564 | -0.5173 | Yes |
| 82 | AASS |  |  | 3539 | -0.564 | -0.5111 | Yes |
| 83 | CELSR2 |  |  | 3550 | -0.570 | -0.5073 | Yes |
| 84 | NCBP2 |  |  | 3588 | -0.598 | -0.5100 | Yes |
| 85 | ZEB1 |  |  | 3589 | -0.598 | -0.5034 | Yes |
| 86 | PCMTD1 |  |  | 3601 | -0.605 | -0.4994 | Yes |
| 87 | ZNF493 |  |  | 3608 | -0.608 | -0.4942 | Yes |
| 88 | AGO1 |  |  | 3611 | -0.609 | -0.4880 | Yes |
| 89 | EPHA3 |  |  | 3616 | -0.614 | -0.4822 | Yes |
| 90 | PHLDA1 |  |  | 3622 | -0.617 | -0.4766 | Yes |
| 91 | GPC2 |  |  | 3633 | -0.630 | -0.4722 | Yes |
| 92 | TFDP2 |  |  | 3637 | -0.634 | -0.4659 | Yes |
| 93 | TRIM2 |  |  | 3688 | -0.672 | -0.4711 | Yes |
| 94 | IP6K2 |  |  | 3689 | -0.672 | -0.4636 | Yes |
| 95 | PCDH9 |  |  | 3707 | -0.691 | -0.4603 | Yes |
| 96 | PREX1 |  |  | 3716 | -0.698 | -0.4546 | Yes |
| 97 | ZKSCAN1 |  |  | 3736 | -0.711 | -0.4515 | Yes |
| 98 | LZTS1 |  |  | 3739 | -0.716 | -0.4441 | Yes |
| 99 | KLHL24 |  |  | 3743 | -0.723 | -0.4368 | Yes |
| 100 | GTF2I |  |  | 3749 | -0.727 | -0.4300 | Yes |
| 101 | TAGLN3 |  |  | 3751 | -0.729 | -0.4222 | Yes |
| 102 | PLCB1 |  |  | 3779 | -0.769 | -0.4205 | Yes |
| 103 | ENC1 |  |  | 3787 | -0.774 | -0.4137 | Yes |
| 104 | SOX4 |  |  | 3797 | -0.786 | -0.4073 | Yes |
| 105 | MAPK10 |  |  | 3798 | -0.787 | -0.3986 | Yes |
| 106 | NREP |  |  | 3805 | -0.794 | -0.3913 | Yes |
| 107 | SCARB2 |  |  | 3811 | -0.798 | -0.3837 | Yes |
| 108 | KIF5C |  |  | 3813 | -0.800 | -0.3751 | Yes |
| 109 | BICD1 |  |  | 3814 | -0.801 | -0.3662 | Yes |
| 110 | MYEF2 |  |  | 3818 | -0.807 | -0.3580 | Yes |
| 111 | CDK2AP1 |  |  | 3850 | -0.844 | -0.3565 | Yes |
| 112 | NKAIN3 |  |  | 3851 | -0.845 | -0.3472 | Yes |
| 113 | DAAM1 |  |  | 3856 | -0.849 | -0.3388 | Yes |
| 114 | MEX3A |  |  | 3866 | -0.867 | -0.3315 | Yes |
| 115 | ROBO2 |  |  | 3879 | -0.899 | -0.3245 | Yes |
| 116 | CHD7 |  |  | 3880 | -0.904 | -0.3145 | Yes |
| 117 | DDAH2 |  |  | 3895 | -0.936 | -0.3077 | Yes |
| 118 | FBLN1 |  |  | 3909 | -0.965 | -0.3003 | Yes |
| 119 | SEL1L3 |  |  | 3919 | -1.001 | -0.2915 | Yes |
| 120 | GPRC5B |  |  | 3937 | -1.032 | -0.2843 | Yes |
| 121 | BAZ2B |  |  | 3947 | -1.046 | -0.2750 | Yes |
| 122 | MEIS3 |  |  | 3951 | -1.060 | -0.2640 | Yes |
| 123 | CUX1 |  |  | 3955 | -1.079 | -0.2529 | Yes |
| 124 | NEDD4L |  |  | 3960 | -1.090 | -0.2418 | Yes |
| 125 | HES6 |  |  | 3961 | -1.092 | -0.2297 | Yes |
| 126 | ZFHX4 |  |  | 3963 | -1.095 | -0.2178 | Yes |
| 127 | SOX11 |  |  | 3977 | -1.147 | -0.2084 | Yes |
| 128 | PGAP1 |  |  | 3981 | -1.164 | -0.1963 | Yes |
| 129 | MMP16 |  |  | 3983 | -1.171 | -0.1836 | Yes |
| 130 | TCF4 |  |  | 3984 | -1.173 | -0.1706 | Yes |
| 131 | MYO10 |  |  | 3990 | -1.193 | -0.1586 | Yes |
| 132 | NFIX |  |  | 4004 | -1.262 | -0.1479 | Yes |
| 133 | NFIB |  |  | 4007 | -1.310 | -0.1339 | Yes |
| 134 | FGD4 |  |  | 4014 | -1.358 | -0.1204 | Yes |
| 135 | KDM5B |  |  | 4016 | -1.364 | -0.1055 | Yes |
| 136 | ZBTB20 |  |  | 4024 | -1.407 | -0.0917 | Yes |
| 137 | MAP2 |  |  | 4051 | -1.673 | -0.0798 | Yes |
| 138 | LINC00461 |  |  | 4068 | -1.907 | -0.0627 | Yes |
| 139 | POU3F2 |  |  | 4071 | -2.024 | -0.0408 | Yes |
| 140 | MEIS2 |  |  | 4073 | -2.041 | -0.0184 | Yes |
| 141 | NFIA |  |  | 4078 | -2.210 | 0.0051 | Yes |
Table: GSEA details [plain text format]

  

Fig 2: RICHARDS\_NOWAKOWSKI\_SCIENCE\_2017\_IPC\_NEN1\_UPREG: Random ES distribution      
 Gene set null distribution of ES for **RICHARDS\_NOWAKOWSKI\_SCIENCE\_2017\_IPC\_NEN1\_UPREG**
